# Influence of Additives on Microstructure and Mechanical Properties of Alumina Ceramics

**DOI:** 10.3390/ma15082956

**Published:** 2022-04-18

**Authors:** Weili Wang, Jianqi Chen, Xiaoning Sun, Guoxun Sun, Yanjie Liang, Jianqiang Bi

**Affiliations:** 1 Key Laboratory for Liquid-Solid Structural Evolution and Processing of Materials, Ministry of Education, School of Materials Science & Engineering, Shandong University, Jinan 250061, China; cjcq1332511377@163.com (J.C.); sava1982@163.com (X.S.); sunguoxun0228@163.com (G.S.); yanjie.liang@sdu.edu.cn (Y.L.); bjq1969@163.com (J.B.); 2 Suzhou Institute of Shandong University, Shandong University, Suzhou 215123, China

**Keywords:** alumina, zirconia, alumina platelets, MXene, mechanical properties

## Abstract

Alumina is one of the most commonly used and researched structural ceramic because of its excellent properties. However, its intrinsic brittleness is the fatal drawback, which hinders it from wider applications. How to improve its fracture toughness as well as the bending strength is always challenging for material researchers. In this paper, alumina matrix composites were fabricated by hot-pressing, in which some additives, including zirconia, alumina platelets, and MXene, were incorporated. The influence of the introduced additives on their microstructure and mechanical properties was investigated. Compare with the monolithic alumina, both bending strength and fracture toughness of all samples were improved greatly. Incorporation of zirconia was beneficial to the mechanical properties due to the phase-transformation strengthening and toughening mechanism. While alumina platelets resulted in high fracture toughness because of the self-toughening of elongated grains. The synergistic effect of alumina platelets and MXene enormously improved the fracture toughness from 2.9 ± 0.3 MPa·m^1/2^ for monolithic alumina to 7.5 ± 0.4 MPa·m^1/2^ for the composite, which was increased by 159%. This work will provide useful references for the fabrication of high-strength and high-toughness alumina ceramics by introducing additives properly.

## 1. Introduction

Alumina (Al_2_O_3_) ceramic possesses excellent merits, such as good wear and corrosion resistance, high hardness, and low price, which make it one of the most intensively studied structural ceramic [1,2]. However, its mechanical properties, especially the fracture toughness is still far below expectations. Strategies have been proposed to improve the brittleness to enlarge its application scope. Among the strengthening and toughening methods, adding additives as reinforcing phases into the Al_2_O_3_ matrix is a useful approach [3]. Up to now, many additives, including metal particle, ceramic particle, whisker, and fiber, have been used to enhance the mechanical properties of Al_2_O_3_ [4,5,6,7,8,9,10,11]. No doubt, zirconia (ZrO_2_) toughened Al_2_O_3_ is a successful example, in which phase transformation of ZrO_2_ plays a vital role in the fracture toughness improvement [12].

With the development of nano materials, 1D and 2D materials are also used to reinforce the mechanical properties of Al_2_O_3_, especially the fracture toughness. For example, the addition of carbon nanotubes (CNTs) for the mechanical property improvement of Al_2_O_3_ are very common in the past years [13,14,15,16]. Zhang et al. fabricated multi-walled CNTs reinforced Al_2_O_3_ composites by pressureless sintering. The composite with small quantities of CNTs exhibited higher flexure strength than pure Al_2_O_3_ [14]. Very recently, Akatsu et al. reinforced Al_2_O_3_ using carbon nanofibers (CNFs) by a layer-by-layer method followed by the densification with SPS. The critical stress intensity factor increased up to about 5.5 MPa·m^1/2^, about 1.5 times larger than that of alumina polycrystals [15]. Graphene is another well-researched additive for Al_2_O_3_ matrix, and many exciting experimental results have been reported [17,18,19,20]. For instance, Graphene oxide/Al_2_O_3_ composites were produced by colloidal method followed with spark plasma sintering. A very low graphene loading led to a 50% improvement on the mechanical properties of Al_2_O_3_ [17]. Liang et al. proposed a molecule-level assembling method to make layer-by-layer stacking structured graphene/Al_2_O_3_ composites. The composite has a dramatically improved fracture toughness, ~3.2 times of the monolithic Al_2_O_3_ [18]. Other 1D and 2D materials, such as boron nitride nanotubes (BNNTs) and boron nitride nanosheets (BNNSs) are also effective reinforcing agents for Al_2_O_3_ ceramic. BNNTs/Al_2_O_3_ composites fabricated by hot pressing displayed excellent ambient and high-temperature mechanical properties due to the pullout and fracture of BNNTs concurrent with the suppression of BNNTs on abnormal grain growth [21]. Additionally, BNNSs/Al_2_O_3_ composites were fabricated by a flocculation method and hot pressing. Compared with the monolith, the bending strength of the composite with 1.0 wt% BNNSs was increased by 58.6% [22].

Recently, MXene, a new type of 2D materials, has attracted more and more attention due to its unique properties [23,24]. Etching layered M_n+1_AX_n_ phases (M is a transition metal; A is a group IIIA or IVA element; X is carbon or nitrogen atoms), which are studied as a kind of high damage-tolerance ceramics for many years [25], and removal of A layer is the general method to obtain MXene [26]. The formula of MXene is written as M_n+1_X_n_T_x_, T_x_ represents the functional groups, such as hydroxyl and fluoride groups, which are introduced on the surface to balance the electric charge during the etching process. Nowadays, MXene becomes a top-research 2D material due to the characteristics of superior mechanical strength, flexibility, and physical/chemical properties, which make it suitable in the application of lithium-ion batteries, supercapacitors, electrocatalysts, electromagnetic interference shielding materials, topological insulators, and so on [23,24]. The extensive achievements verified that MXene is a promising filler for improve the properties of metals, polymers as well as ceramics. For example, ultrathin nanosheets of Ti_3_Si_0_._75_Al_0_._25_C_2_ MAX was added into poly(methylmethacrylate), the composite showed excellent thermal and mechanical properties, including improved glass-transition temperature, thermal conductivity, Young’s modulus, and decreased thermal expansion [27]. However, few papers have reported the reinforcing effect of MXene on the properties of ceramics. Feng et al. reported the addition of MXene into Al_2_O_3_ matrix produced a positive effect on the mechanical properties because of the grain growth restriction, matrix densification and cracks deflection [28]. The limited researches are inadequate to elevate the promising application of MXene in ceramics [28,29]. Therefore, experiments and related studies on the mechanisms should be carried out, with increasing urgency, for the effective utilization of MXene in ceramic matrix.

In this study, a simple ball-milled mixing approach combined hot-pressing was used to fabricate Al_2_O_3_ matrix composites. Several additives, including ZrO_2_, Al_2_O_3_ platelets and Ti_3_C_2_T_x_ (a typical MXene) were introduced. These additives affected the mechanical properties of the Al_2_O_3_ ceramics significantly. In general, improvement in mechanical properties including bending strength and fracture toughness were demonstrated. Microstructural analysis was also performed to investigate the mechanism of mechanical enhancement in-depth.

## 2. Experimental

The MXene was synthesized by a common and facile HF etching method. Typically, 10 g Ti_3_AlC_2_ powders were added into 100 mL HF solution and magnetically stirred for 72 h at 25 °C. The etched powders were rinsed with deionized water and ethanol for several times, and dried in an oven at 60 °C for 24 h. Then the collected powders were put into ethanol for sonication for 3 h. After dried at 60 °C for 24 h, Ti_3_C_2_T_x_ was obtained and kept in vacuum. Before ball mill mixing with other powders, the Ti_3_C_2_T_x_ MXene was dispersed in 200 mL ethanol by magnetic stirring vigorously for 1 h followed by sonication for 3 h, respectively.

The α-Al_2_O_3_ powder (Hang Zhou Veking New Material Co., Ltd., Hangzhou, China, 500 nm) was used as the basis material to fabricate Al_2_O_3_ ceramic and its composites. α-Al_2_O_3_ platelets (Ronaflair white sapphire, Merck), yttrium stabilized zirconia powder (3Y-ZrO_2_, Hang Zhou Veking New Material Co., Ltd., Hangzhou, China, 100 nm), and the synthesized MXenes were used as the additives to improve Al_2_O_3_ ceramics’ mechanical properties. The above-mentioned materials were accurately weighed, mixed with ethanol and ball milled for 8 h. Then the slurries were placed in oven to remove ethanal at 80 °C for 24 h. The dried powder was collected and put into a graphite mold with an inner diameter of 42 mm for hot-pressing (High-Multi 5000, Fuji Dempa Kogyo Co., Ltd., Osaka, Japan). The ceramic samples were fabricated at a temperature of 1500 °C under a pressure of 30 MPa in an argon atmosphere for 1 h.

After polishing, the density of the samples was measured via the Archimedes method in distilled water. The density of 3.97 g/cm^3^, 5.90 g/cm^3^ and 4.00 g/cm^3^ was adopted for Al_2_O_3_, ZrO_2_ and MXene [30] as the theoretical density, respectively. Subsequently, the samples were grounded by a diamond grinding wheel and cut into bars for mechanical property measurement on an CMT6203 universal testing machine (MTS Systems (China) Co., Ltd., Shenzhen, China). Before test, all the surfaces of test bars were polished with B_4_C abrasive finely to remove the scratches arising from the grounding the cutting process. Moreover, the edges of the bars were also chamfered to minimize stress concentration originating from the defects. The bending strength was measured by the three-point bending method using the 3.0 mm (width) × 4.0 mm (thickness) × 30.0 mm (length) bar specimens. The span length and load speed were 20.0 mm and 0.5 mm/s, respectively. The single-edge notched beam (SENB) method was employed for fracture toughness test. The bar specimens with the dimension of 2.0 mm (width) × 4.0 mm (thickness) × 30.0 mm (length) as well as a notch (0.3 mm (width) × 2.0 mm (depth)) was introduced in the center. The span length of 20.0 mm and crosshead speed of 0.05 mm/s were used. Generally, four specimens of each sample were used for bending strength and fracture toughness testing, and the averages were taken as the values of the mechanical properties.

A Rigaku D/max-RA X-ray diffractometer (XRD) with Cu Kα X-ray source was employed to conduct phase analysis of the sintered samples. The microstructural observation was carried out using a Hitachi SU-70 type thermal field emission scanning electron microscope (FESEM). The crack paths in the fractured samples were observed in an optical microscope (LW600LT, Shanghai Cewei Optoelectronic Technology Co., Ltd., Shanghai, China).

## 3. Results and Discussion

To investigate the influence of the additives on the bending strength and fracture toughness of the Al_2_O_3_ matrix composites, samples with different compositions were prepared. The number and the exact composition of samples ae shown in Table 1.

**Figure 1:**
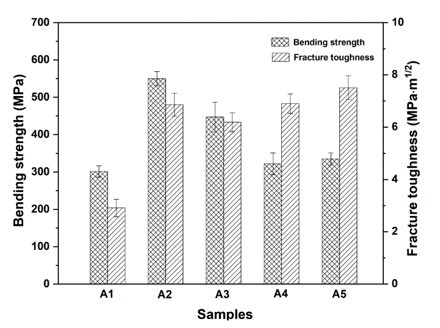
Mechanical properties of the samples.

**Figure 2:**
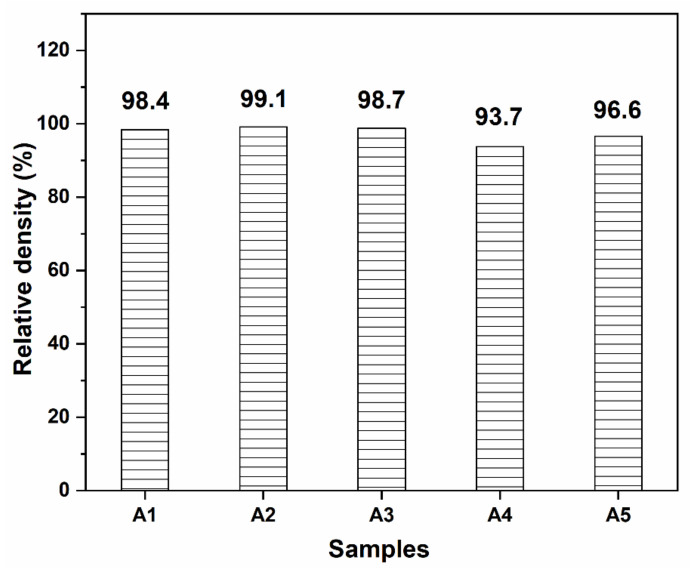
Relative densities of the samples.

The variation tendency of mechanical properties including bending strength and fracture toughness is displayed in Figure 1. It is found that all the additives exerted notable effect on the mechanical properties. When 3Y-ZrO_2_ is added into the matrix (A2), both of bending strength and fracture toughness are increased greatly. Compare with the monolithic Al_2_O_3_ ceramic (A1), the bending strength and fracture toughness increase from 301.6 MPa and 2.91 MPa·m^1/2^ to 549.9 MPa and 6.86 MPa·m^1/2^, which are improved by 82% and 136%, respectively. However, the addition of MXene does not increase the mechanical properties furtherly. By contrast, both bending strength and fracture toughness decrease to 447.2 MPa and 6.19 MPa·m^1/2^ (A3). Although the mechanical properties decrease, both bending strength and fracture toughness are still much higher than the pure Al_2_O_3_. In comparison with A1, A4, and A5, the introduction of Al_2_O_3_ platelet is also beneficial to the mechanical properties, especially the fracture toughness. Compare with A1, A4 with the addition of 20.0 wt% Al_2_O_3_ platelet possesses high fracture toughness (6.90 MPa·m^1/2^) as well as a slight increase of bending strength. Amazingly, when MXene is incorporated simultaneously, the coupling effect of Al_2_O_3_ platelet and MXene in the improvement of fracture toughness is very noticeable. The fracture toughness of A5 reaches 7.51 MPa·m^1/2^, which is increased by 159%. In addition, all the samples have relatively high relative densities, as shown in Figure 2. The samples containing Al_2_O_3_ platelet possess lower relative densities than those of other samples.

**Figure 3:**
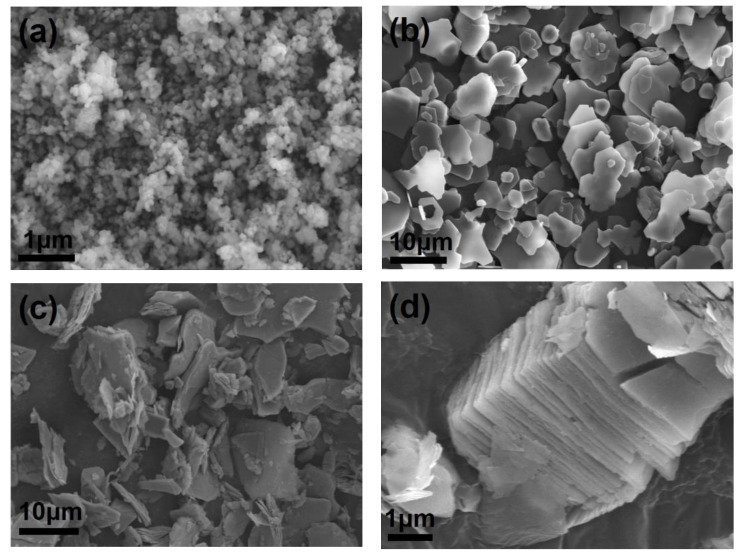
SEM images of additives, (**a**) 3Y-ZrO_2_; (**b**) Al_2_O_3_ platelet; (**c**,**d**) Ti_3_C_2_T_x_ MXene.

SEM observation is conducted to investigate the morphology of the additives, as shown in Figure 3. It can be seen from Figure 3a that the particle size of 3Y-ZrO_2_ is ~100 nm as the supplier claims. Al_2_O_3_ platelets are plate-like with irregularly morphology in Figure 3b. Ti_3_C_2_T_x_ MXene can be found in Figure 3c,d. The MXene are platelet shape with different particle size (Figure 3c), and the layered structure can be observed clearly in Figure 3d. The Ti_3_C_2_T_x_ layer stacks together to form a unique accordion structure. The disparate morphology and structure of additives play an important role in the mechanical property improvement as mentioned in Figure 1.

**Figure 4:**
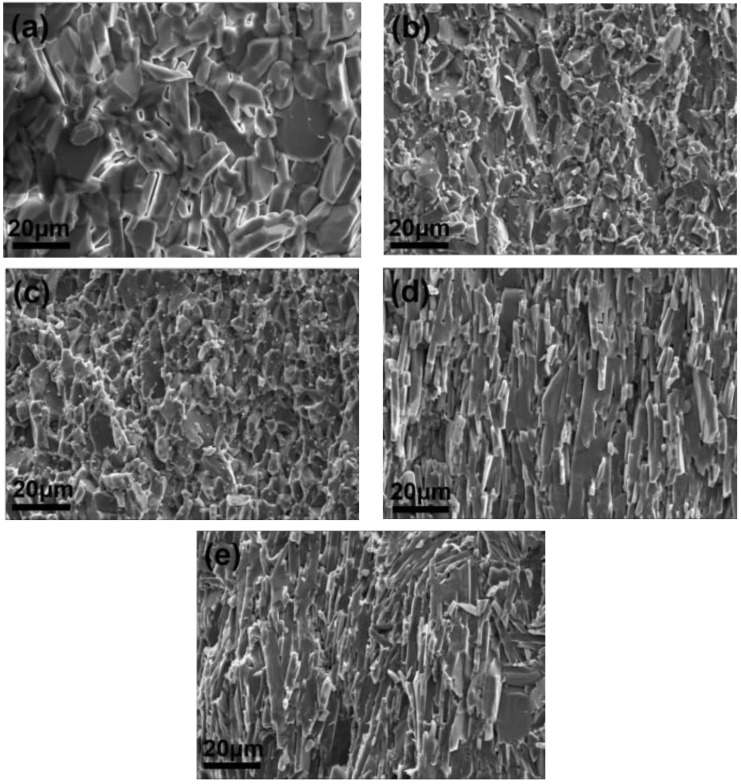
SEM images of fracture surface, (**a**) A1; (**b**) A2; (**c**) A3; (**d**) A4; (**e**) A5.

The fracture surface images were used for comparative analysis of the grain size evolution depending on additives incorporated in alumina matrix. As displayed in Figure 4, the fracture surface morphology varies greatly with the additives. It is revealed that the grain size distribution is not uniform for the monolith Al_2_O_3_ (A1, Figure 4a). However, when ZrO_2_ is added, the grain size become smaller (A2, Figure 4b). For A3 as shown in Figure 4c, the grains are still very small, and the edges after fractured are much clearer. Notably, the number of large grains becomes less in comparison with A2. When Al_2_O_3_ platelet is added, surprisingly, the Al_2_O_3_ grains are elongated, with a very high length-width ratio (Figure 4d,e). In comparison with A5, the layered structure of A4 is more regular. The thickness of the elongated Al_2_O_3_ grains in A5 is thinner, and their arrangement is more disorder. Based on the above observation, the addition of additives has a great influence on the morphology evolution of the samples, thus affect their mechanical properties.

Compare with A1, A2 possesses much better mechanical properties. The addition of ZrO_2_ has a little effect on the relative density in Figure 2. However, the low-magnification SEM images in Figure 4b,c shown that the addition of ZrO_2_ results in finer grains. It is well-known that a Hall–Petch relationship (Equation (1)) exists in metallic materials, which reveals the relationship between grain size and yield strength.

**Figure 5:**
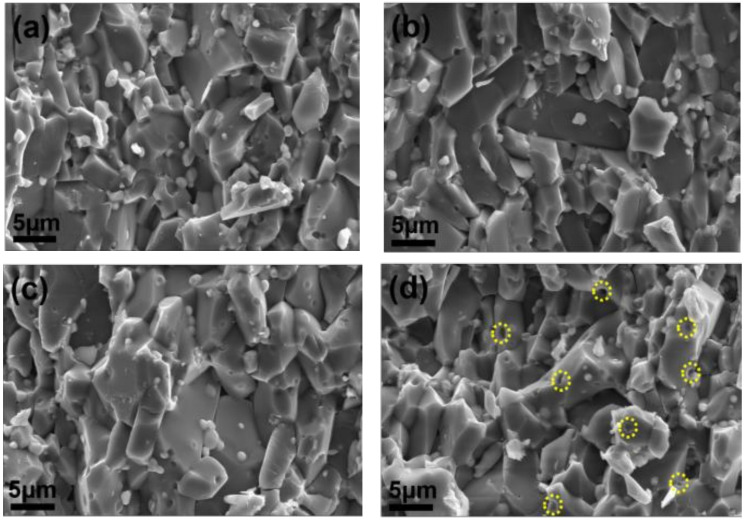
SEM images of fracture surface, (**a**,**b**) A2; (**c**,**d**) A3.

*σ_b_ =σ*_0_ + *κ·d*^−1/2^ (1)

where *σ*_0_ and *κ* are material constants and independent of grain size, and *d* is grain size. According to the classic Hall–Petch relationship, grain refinement contributes to enhance strength of metals [31]. In Al_2_O_3_ and its CNTs reinforced composite, a similar relationship between grain size and bending strength exists, which means that refined grains are ascribed to bending strength improvement [32,33,34]. Therefore, the addition of ZrO_2_ leads to grain refinement, and thus to a degree of bending strength enhancement. However, phase transformation strengthening and toughening mechanism of ZrO_2_ is believed the main reason why the bending strength and fracture toughness are improved significantly. As well-known, the metastable *t*-ZrO_2_ in the partially stabilized ZrO_2_ is the main strengthening and toughening factor during the samples fracture [12]. In the stress field of the crack, the transformation of *t*-ZrO_2_→*m*-ZrO_2_ accompanied by a volume dilatation [3]. The volume effect and shape effect originating from the transformation will absorb energy, leading to a great enhancement of mechanical properties. When MXene is added in A3, the mechanical properties decrease unexpectedly. The SEM images in Figure 5 can give some explanations. When only ZrO_2_ is added, the ZrO_2_ grains are mainly located at Al_2_O_3_ grain boundaries, and the grains grow up after sintering. It is noticeable that the fracture mode of the sample (A2) is mainly trans-granular fracture as shown in Figure 5a, which is believed to consume more energy during the fracture process. Additionally, the inserted elongated Al_2_O_3_ grains are also helps to improve the mechanical properties due to more energy consumption (Figure 5b). However, when MXene is added combine with ZrO_2_, most of the elongated Al_2_O_3_ grains are missing as shown in Figure 5c. Meanwhile, the fracture mode is mainly inter-granular fracture. Compare with A2, it can be observed in Figure 5d that the growth of ZrO_2_ grains are inhibited. In particular, many ZrO_2_ grains are dropped out, and leaving holes on the surface during the fracture process (yellow circles in Figure 5d). Therefore, the mechanical properties of A3 decrease. Anyway, A3 still possesses good bending strength and fracture toughness, about 48% and 113% higher than A1, respectively.

**Figure 6:**
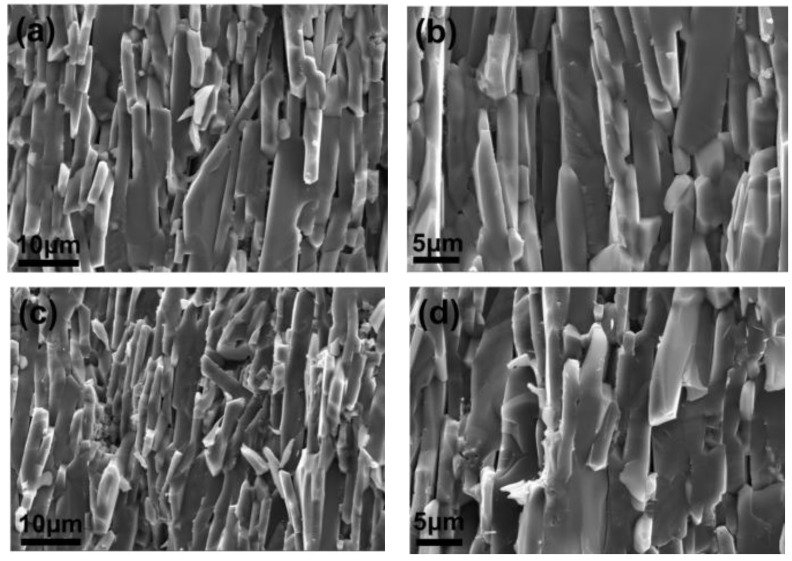

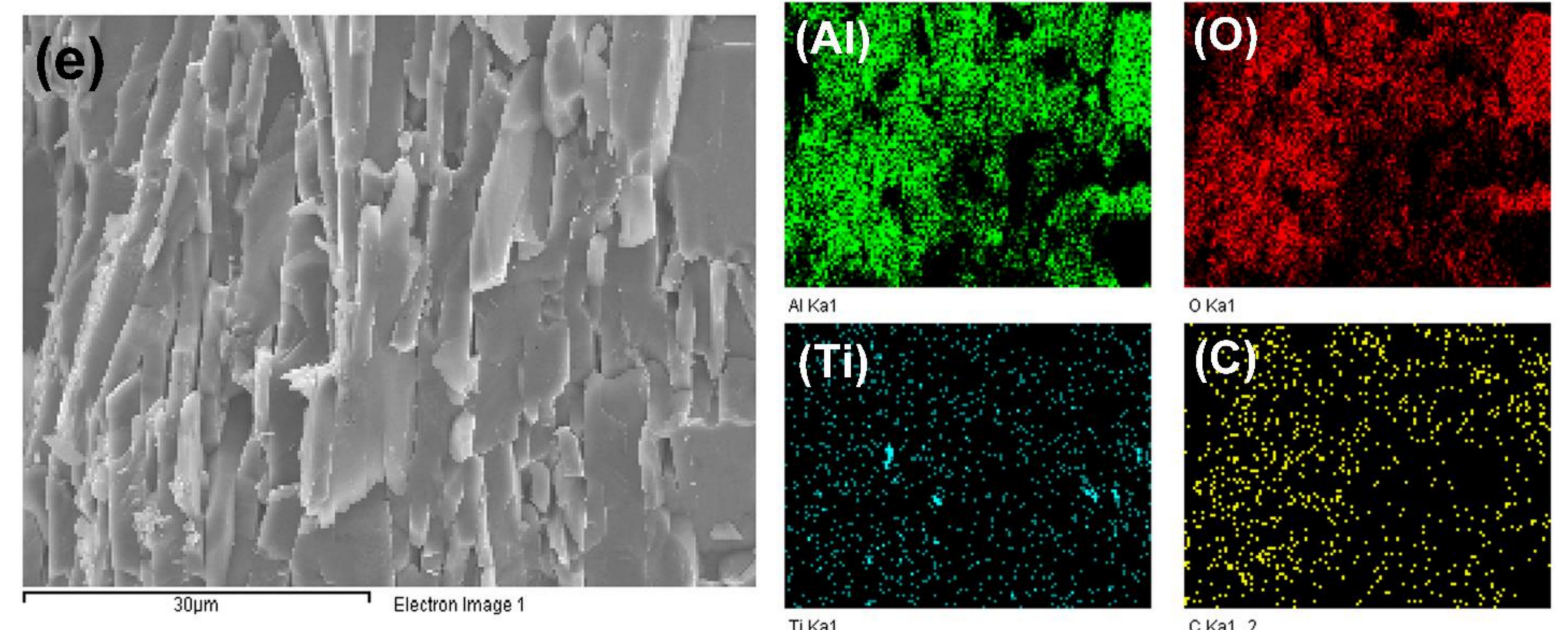
SEM images of fracture surface, (**a**,**b**) A4; (**c**,**d**) A5 and (**e**) ESD analysis of A5.

**Figure 7:**
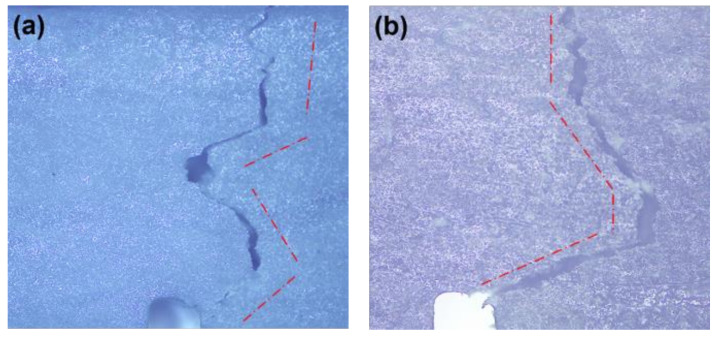
Optical images of crack path, (**a**) A4; (**b**) A5.

In comparison to A1, A4, and A5, some interesting results have also been found. Although the bending strength is increased slightly, fracture toughness is improved significantly (Figure 1). When only Al_2_O_3_ platelet is added, the fracture toughness is increased by 137%, which is from 2.91 MPa·m^1/2^ (A1) to 6.90 MPa·m^1/2^ (A4). The simultaneous addition of Al_2_O_3_ platelet and MXene is expected to produce exciting mechanical properties. It is surprising that the fracture toughness of 7.51 MPa·m^1/2^ is achieved. As many literatures report, the addition of some additives is favorable to anisotropic grain growth behavior, thus leading to self-reinforcement in Al_2_O_3_ ceramic [35,36,37]. Although no liquid-formation impurities were introduced, the platelet shape of Al_2_O_3_ platelet still induced the abnormal grain growth, and forming a layered structure as shown in Figure 6. When using Al_2_O_3_ platelet as additive alone (A4), the layered Al_2_O_3_ grains arrange regularly (Figure 6a), besides, pores can be observed on the surface as exhibited in Figure 6b, causing a decrease of relative density (Figure 2). With the simultaneous addition of Al_2_O_3_ platelet and MXene, the Al_2_O_3_ layers become thinner and disordered (Figure 6c). The MXene distributes homogenously on the surface as the EDS mapping displayed in Figure 6e. Particularly, when the MXene platelet inserts into the Al_2_O_3_ layers, they will inhibit grain growth at one direction, and fill in the pore between layers (Figure 6d). Therefore, A5 has higher relative density than A4. What is more, the disordered layers are conducive to crack deflection and layer lock, thus are conducive to energy consumption and mechanical property enhancement. The zigzag crack path can be seen clearly for both A4 and A5 in Figure 7. Compare with A4 (Figure 7a), A5 displays a more tortuous cracks propagation path (Figure 7b), so it possesses the highest fracture toughness in this work. In general, the above additives play a positive effect on the mechanical properties of Al_2_O_3_ ceramic, either bending strength or fracture toughness. The results will guide the design and fabrication of high-strength and high-toughness alumina ceramics by introducing proper additives. Meanwhile, it can provide refence to investigate the effect of MXene on the properties of ceramic matrix composites.

## 4. Conclusions

In summary, Al_2_O_3_ matrix composites with different additives were fabricated by hot-pressing. The employment of the additives had great influences on the microstructure and mechanical properties of Al_2_O_3_ ceramics. The experimental results showed that the addition of ZrO_2_ refined the Al_2_O_3_ grain size; while Al_2_O_3_ platelet induced the anisotropic grain growth, leading to a layered structure in Al_2_O_3_. Meanwhile, the incorporation of MXene refined the grains furtherly, leading to smaller Al_2_O_3_ and ZrO_2_ grains in A3 and thinner Al_2_O_3_ layers in A5. Regarding mechanical properties, the incorporation of ZrO_2_ was beneficial to the mechanical properties due to the phase-transformation strengthening and toughening mechanism. By contrast, the introduction of alumina platelets resulted in high fracture toughness because of the self-toughening of elongated grains. Encouragingly, the synergistic effect of Al_2_O_3_ platelets and MXene improved the fracture toughness enormously, from 2.9 ± 0.3 MPa·m^1/2^ for monolithic alumina to 7.5 ± 0.4 MPa·m^1/2^ for the composite, which was increased by 159%.

## Figures and Tables

**Table 1:** Composition of the samples.

No.	3Y-ZrO_2_ Content (wt%)	Al_2_O_3_ Platelet Content (wt%)	MXene Content (wt%)
A1	—	—	—
A2	5.0	—	—
A3	5.0	—	1.0
A4	—	20.0	—
A5	—	20.0	1.0

## Data Availability

All the supporting and actual data are presented in the manuscript.
